# Root form and canal morphology of maxillary first premolars of a Yemeni population

**DOI:** 10.1186/s12903-018-0555-x

**Published:** 2018-05-31

**Authors:** Elham M. Senan, Hatem A. Alhadainy, Thuraia M. Genaid, Ahmed A. Madfa

**Affiliations:** 1 Restorative and Prosthodontic Department, College of Dentistry, University of Science and Technology, Sana’a, Yemen; 2 Department of Dentistry, University of Alberta, Edmonton, Canada; 3 Department of Conservative Dentistry, Faculty of Dentistry, Tanta University, Tanta, Egypt; 4 Department of Conservative Dentistry, Faculty of Dentistry, Thamar University, Dhamar, Yemen; 5 Department of Endodontics, Faculty of Dentistry, Tanta University, Tanta, Egypt

**Keywords:** Maxillary first premolar, Root canal morphology, Yemeni population, Clearing technique

## Abstract

**Background:**

The purpose of this study was to investigate variations in the root canal systems of permanent maxillary first premolars in a Yemeni population using a clearing technique.

**Methods:**

Two hundred fifty permanent maxillary first premolar teeth extracted from Yemeni individuals were collected. A small hole in the center of the occlusal surface of each tooth was prepared and pulp tissue was removed by immersion in 5.25% sodium hypochlorite. Teeth were stored in 5–10% nitric acid solution for 5–6 days. Next, teeth were rinsed, dried, and dehydrated using ascending concentrations of ethanol (70, 95, and 100%) successively for 12 h each. Waterproof black ink was injected into the dried dehydrated teeth. Stained teeth were then rendered clear by immersion in methyl salicylate solution (98%) until evaluation. Root canal morphology of each tooth was then examined.

**Results:**

54.8% of teeth were single-rooted, while 44.4% were double-rooted and only 0.8% had three separated roots. The most common canal system configuration was Vertucci type IV (55.6%). Eight specimens of the single-rooted premolars (3.2%) had new canal configurations that have not been recognized in previous published studies. Accessory canals and inter-canal communications were detected in a total of 52.8 and 34.4% of the specimens, respectively. The apical foramen was located centrally to the apex in 84.9% and apical deltas were found in 13.2% of the studied sample.

**Conclusions:**

Yemeni permanent maxillary first premolars are mainly single-rooted and predominantly present Vertucci type IV canal morphology. The finding of additional canal configurations in this study is low but should be kept in mind when performing endodontic therapy for these teeth.

## Background

Root canal treatment is an essential part of comprehensive, quality dental care [1]. Successful endodontic treatment depends on complete root canal cleansing and shaping, three-dimensional hermetic root canal system obturation, and well-fitting coronal restorations with no leakage [2]. However, lack of thorough knowledge about teeth internal anatomy is one of the main reasons for treatment failure in endodontics. Thus, dental practitioners must be familiar with root canal morphology of teeth to be treated. Such knowledge can aid in localization and negotiation of canals, as well as their subsequent management [3]. Unfortunately, root canal morphology varies greatly among different populations and even in different individuals within the same population. Therefore, an accurate knowledge of root canal morphology and its anatomical variations is essential for a successful root canal treatment [4].

Maxillary first premolar represents one of the most difficult teeth to be treated endodontically. A number of studies exhibited great variations in root anatomy and root canal morphology [5–13]. These variations in number and type of root canals are probably some of the most widely described anomalies in the literature. The presence of two canals must be considered normal [14, 15], but racial differences in the root canal morphology of maxillary first premolar have been established [14–17].

Numerous studies have dealt with the evaluation of root canal morphology among different populations using various techniques, such as radiographs, decalcification, sectioning, replication and computerized-aided techniques [11–13, 18–21]. Of all these techniques, teeth clearing technique has considerable value in studying the morphology of root canal system. This is because clearing technique provides a three-dimensional view of the pulp cavity in relation to the exterior of teeth and allows a comprehensive examination of the pulp chamber and root canal system [4].

Clinically, it is important to identify the root and canal morphology prevalent in a population to reduce errors during root canal treatment. However, no study in Yemen has yet investigated the incidence of root canal configurations in any tooth. Therefore, the purpose of this study was to evaluate root and canal morphology of permanent maxillary first premolar teeth in a Yemeni population using a clearing technique.

## Methods

The present study was approved by the Medical Ethics Committee (MEC) of Faculty of Medicine and Health Sciences at University of Science and Technology, Sana’a, Yemen (MECA NO.: 2016/13). Two hundred fifty recently extracted maxillary first premolars were collected from Yemeni patients attending various orthodontic clinics in Sana’a city. All teeth were identified at the time of extraction as maxillary first premolars from Yemeni patients attending orthodontic clinics. All patients signed consents acknowledging that their teeth will be used in the study. Teeth sample was collected in one and a half year.

Gender of the patient was not recorded and the age range was 20–45 years. The extracted teeth were thoroughly washed and cleaned to remove blood, saliva, or debris. They were then placed in 5.25% sodium hypochlorite solution for 30 min to remove organic debris from the surface. If there was calculus, it was removed using scaler (miniPiezon®, Electro Medical Systems EMS, Nyon, Switzerland). The cleaned specimens were then saved in 10% formalin solution (Oxford Laboratory, Mumbai, India) until further investigation was carried out [13].

External root morphology was determined visually and the findings were recorded. The specimens were classified into three groups based on the forms and number of roots as follows: single-rooted, double-rooted and three-rooted premolars. After recording the external root morphology of the specimens, a small hole in the center of the occlusal surface of each tooth was prepared as access to the pulp cavity. The specimens were then immersed in 5.25% sodium hypochlorite for 4 h to remove pulpal tissues. They were then rinsed under running tap water for 2 h and dried overnight. Afterwards, the specimens were decalcified with 10% nitric acid (Gainlad Chemical Co., Clwyd, UK) for 3 days followed by 5% nitric acid for 2–3 days at room temperature. The nitric acid solution was changed daily and agitated once a day to speed the process of decalcification. Then, the specimens were tested for softness by inserting a needle into the coronal region [13]. Decalcified specimens were then rinsed thoroughly and stored in water overnight and were bench-dried for 3 h. They were dehydrated in successive solutions of 70, 95 and 100% ethanol (Scharlau Co., European Union); each for 12 h. Once the dehydration process was completed, teeth were allowed to bench-dry for 2 h.

To clearly view the root canal system, waterproof black ink (Sanford rotring GmbH, Hamburg, Germany) was coronally injected into the pulp chambers using an endodontic irrigation syringe with a 27 gauge needle (BU Kwang Medical Inc., Seoul, Korea) until the ink was seen out through the apical foramen. Excess ink was then removed from the surface of the specimens with gauze soaked in ethanol. The stained specimens were then bench-dried for 4 h. Finally, transparency was achieved by placing the specimens in 98% methyl salicylate (ACROS Organics, New Jersey, USA).

Standardized pictures of the transparent cleared teeth were obtained by digital photographing both mesio-distally and bucco-lingually with a fixed distance (10 cm) and zoom (× 2.5). Photographs were taken with a light-illuminated white paper wet with methyl salicylate solution as a background. Evaluation of cleared teeth images was performed independently by two endodontists, each with an experience of more than five years. This was done after calibration to Vertucci canal types’ classification. Inter-examiner agreement was evaluated using Kappa test on SPSS. The following observations were recorded: (i) number and type of root canals; (ii) presence and location of both accessory canals and inter-canal communications (ICCs); (iii) location and number of apical foramina and (iv) presence of apical deltas.

## Results

### Morphology and number of roots

**Fig. 1:**
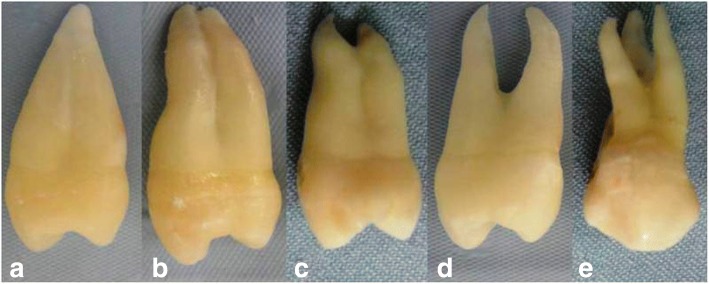
Clinical photographs showing variations in number of root and morphology in permanent maxillary first premolars.**a** One root with single tip, (**b**) one root with double tips, (**c**) fused two roots, (**d**) separated two roots and (**e**) separated three roots

Of the 250 maxillary first premolars studied, 137 teeth had one root (54.8%), whilst 36.4% were single-tipped root apex and the rest (18.4%) had double-tipped root apex. Of the 111 (44.4%) double-rooted premolars, 29.2% had two separated roots and 15.2% had two fused roots (they exhibited bifurcation in the apical third). Two premolars of the study sample (0.8%) had three separated roots (Fig. 1).

### Number and type of root canals

**Fig. 2:**
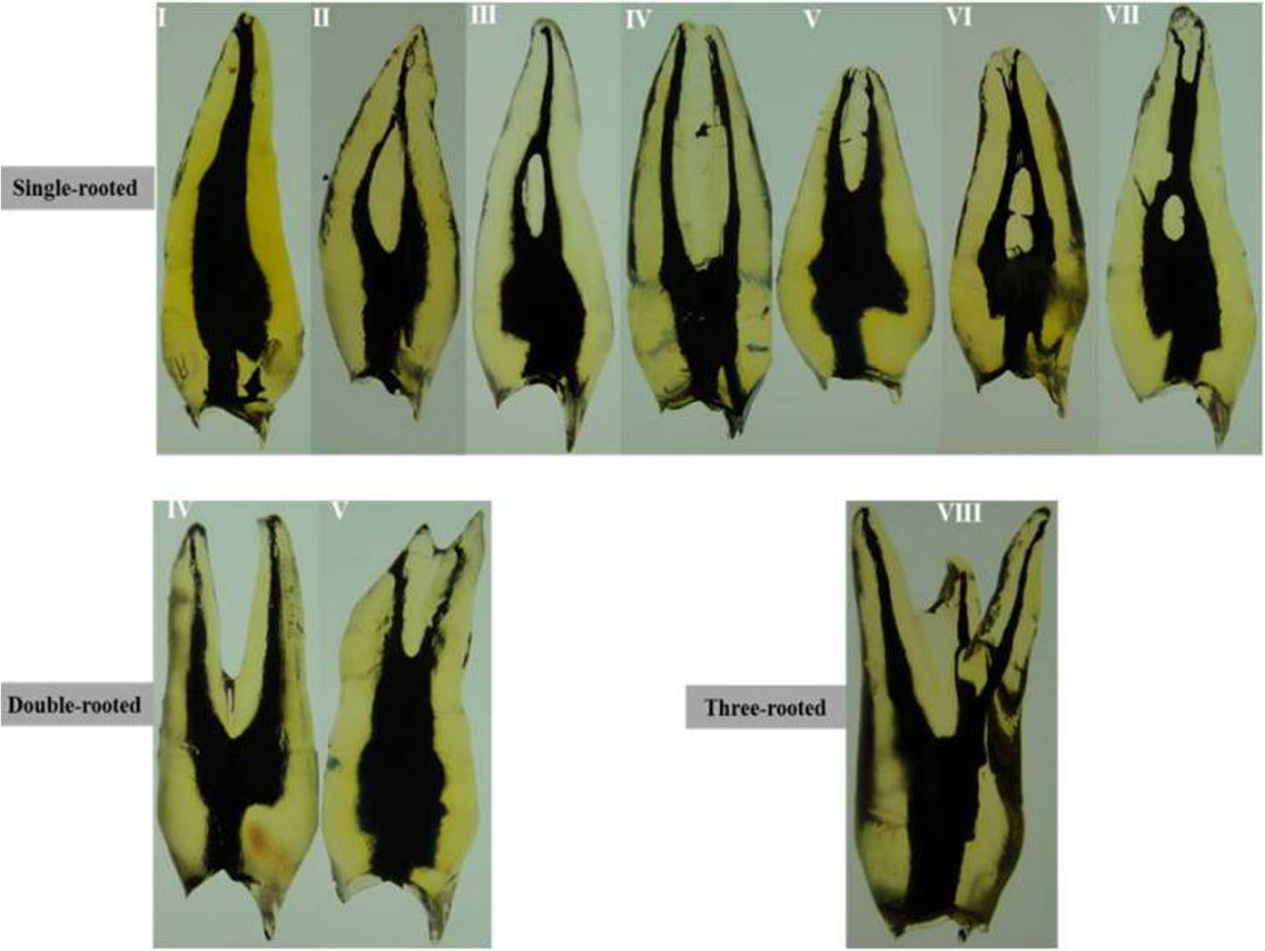
Cleared teeth demonstrating Vertucci’s canal configurations of Yemeni permanent maxillary first premolars

**Fig. 3:**
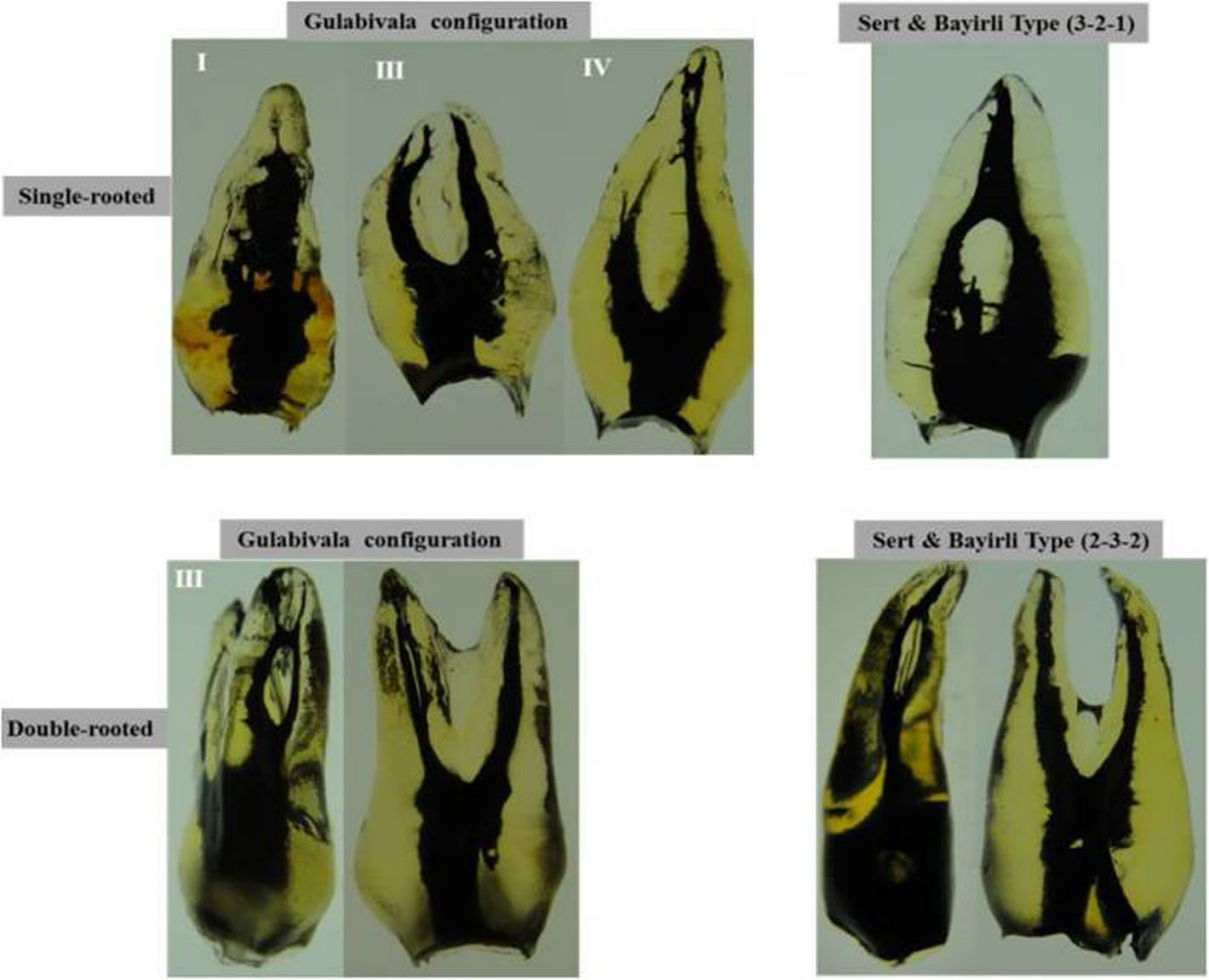
Cleared teeth demonstrating supplemental canal configurations of Yemeni permanent maxillary first premolars

**Fig. 4:**
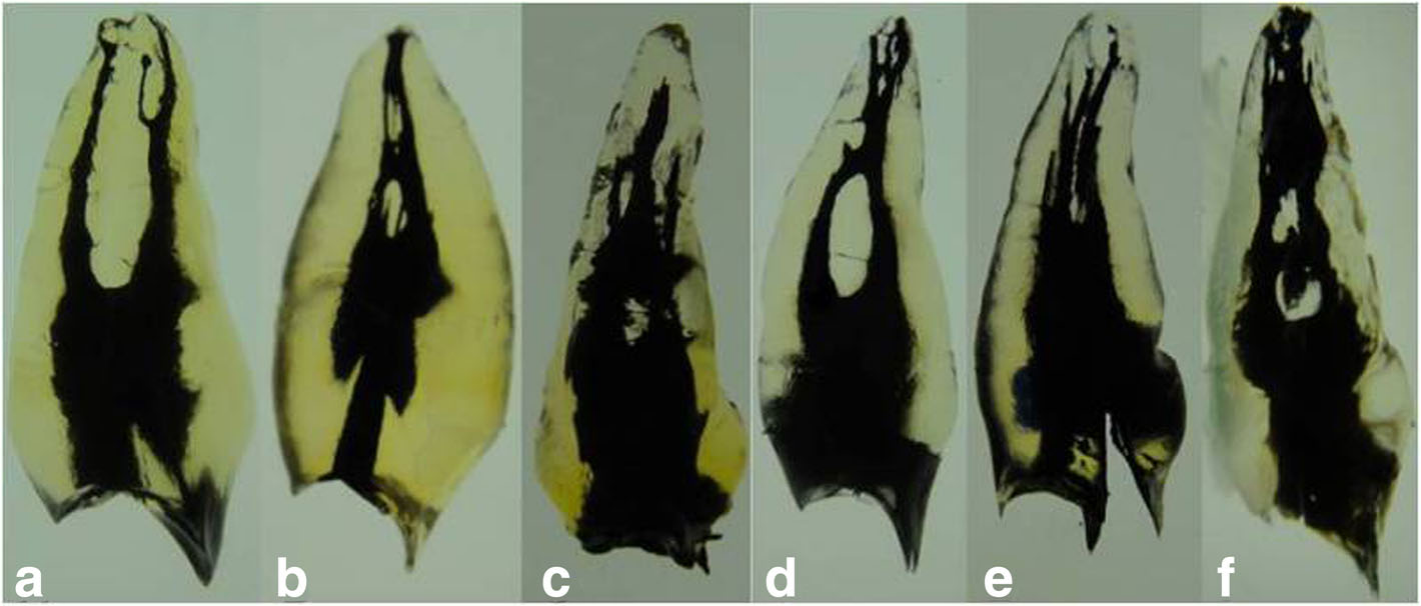
Cleared teeth showing new canal types of Yemeni permanent maxillary first premolars. **a** Type (1–2-3), (**b**) Type (1–2–1-2-1), (**c**) Type (1–2–1-3-2), (**d**) Type (1–2–1-2-3-2), (**e**) Type (1–3-4), and (**f**) Type (1–2–1-3-2)

The data for number and type of root canal system were revealed in Table 1. Single-rooted premolars demonstrated a wide variation of canal configurations (Figs. 2, 3,4). Seventy-four of the single-rooted specimens had one canal (54.1%) of either Vertucci type I (24.1%), III (14.6%), V (8.8%) or VII (6.6%) configuration, while 35.7% (*n* = 49) of the specimens had two canals of either type II (8.0%), IV (24.8%) or VI (2.9%) configuration. In addition, four cases of the single-rooted premolars had two canals (2.9%) of either Gulabivala type III (2.2%), or IV (0.7%) configuration. Furthermore, two cases of the single-rooted premolars had three canals (1.5%) of either Gulabivala type I (0.7%), or (3–2-1) Sert & Bayirli (0.7%) configuration (Table 1). Moreover, eight specimens of the single-rooted premolars had one canal (3.2%) with new canal configurations that have not been recognized in previous studies (Table 1, Fig. 4).

**Table 1:** The different anatomical features of Yemeni permanent maxillary first premolars

Features	Single-rooted *n (%)*	Double-rooted *n (%)*	Three-rooted *n (%)*	Total *n (%)*
Vertucci classification				
Type I (1)	33 (24.1%)	0 (0.0%)	0 (0.0%)	33 (13.2%)
Type II (2–1)	11 (8.0%)	0 (0.0%)	0 (0.0%)	11 (4.4%)
Type III (1–2-1)	20 (14.6%)	0 (0.0%)	0 (0.0%)	20 (8.0%)
Type IV (2)	34 (24.8%)	105 (94.6%)	0 (0.0%)	139 (55.6%)
Type V (1–2)	12 (8.8%)	2 (1.8%)	0 (0.0%)	14 (5.6%)
Type VI (2–1-2)	4 (2.9%)	0 (0.0%)	0 (0.0%)	4 (1.6%)
Type VII (1–2–1-2)	9 (6.6%)	0 (0.0%)	0 (0.0%)	9 (3.6%)
Type VIII (3)	0 (0.0%)	0 (0.0%)	2 (100%)	2 (0.8%)
Gulabivala classification				
Type I (3–1)	1 (0.7%)	0 (0.0%)	0 (0.0%)	1 (0.4%)
Type III (2–3)	3 (2.2%)	2 (1.8%)	0 (0.0%)	5 (2.0%)
Type IV (2–1–2-1)	1 (0.7%)	0 (0.0%)	0 (0.0%)	1 (0.4%)
Additional types				
Type (3–2-1) Sert & Bayirli	1 (0.7%)	0 (0.0%)	0 (0.0%)	1 (0.4%)
Type (2–3-2) Sert & Bayirli	0 (0.0%)	2 (1.8%)	0 (0.0%)	2 (0.8%)
New types				
Type (1–2-3)	2 (1.5%)	0 (0.0%)	0 (0.0%)	2 (0.8%)
Type (1–2–1-2-1)	2 (1.5%)	0 (0.0%)	0 (0.0%)	2 (0.8%)
Type (1–2–1-3-2)	1 (0.7%)	0 (0.0%)	0 (0.0%)	1 (0.4%)
Type (1–2–1-2-3-2)	1 (0.7%)	0 (0.0%)	0 (0.0%)	1 (0.4%)
Type (1–3-4)	1 (0.7%)	0 (0.0%)	0 (0.0%)	1 (0.4%)
Type (1–2–1-3-2)	1 (0.7%)	0 (0.0%)	0 (0.0%)	1 (0.4%)
No. accessory canals				
Accessory canals present	65 (47.4%)	66 (59.5%)	1 (50%)	132 (52.8%)
Accessory canals absent	72 (52.6%)	45 (40.5%)	1 (50%)	118 (47.2%)
Accessory canals in				
Cervical third	4 (5.4%)	9 (11.7%)	0 (0.0%)	13 (8.5%)
Middle third	16 (21.6%)	29 (37.7%)	1 (50%)	46 (30.1%)
Apical third	54 (73.0%)	39 (50.6%)	1 (50%)	94 (61.4%)
No. ICCs				
ICCs present	63 (46.0%)	23 (20.7%)	0 (0.0%)	86 (34.4%)
ICCs absent	74 (54.0%)	88 (79.3%)	2 (100%)	164 (65.6%)
ICCs in				
Cervical third	30 (33.0%)	13 (46.5%)	0 (0.0%)	43 (36.2%)
Middle third	39 (42.9%)	9 (32.1%)	0 (0.0%)	48 (40.3%)
Apical third	22 (24.1%)	6 (21.4%)	0 (0.0%)	28 (23.5%)

On the other hand, the double-rooted specimens exhibited Vertucci types IV and V in 105 (94.6%) and 2 (1.8%), respectively (Table 1, Figs. 2 and 3). In addition, both Gulabivala type III (1.8%) and (2–3-2) Sert & Bayirli (1.8%) configurations were found in two specimens. Of the three-rooted specimens, both cases exhibited three canals (Table 1, Fig. 2).

### Accessory canals, inter-canal communications, apical foramina and deltas

Accessory canals were detected in a total of 132 (52.8%) of the specimens (Table 1). They were more frequently observed in the apical third as compared to cervical and middle thirds of the roots. Inter-canal communications (ICCs) were present in 86 (34.4%) of the specimens. ICCs were more prevalent in the single-rooted group (25.2%) compared to the double-rooted (9.2%) premolars (Table 1). The apical foramen was located centrally to the apex in 84.9% of the studied sample. Out of all the canals; canals exiting in single foramina were 77.5%; whereas, 19.7% exited in two separate foramina. Only in 2.8% of the specimens, three apical foramina were present (Table 2). Apical deltas were found in 33 specimens (13.2%), of which 29 (11.6%) were in single-rooted group and 4 (1.6%) were in double-rooted premolars. However, apical deltas were not detected in the three-rooted premolars (Table 2).

**Table 2:** Distribution of apical foramina and deltas of Yemeni permanent maxillary first premolars

Features	Single-rooted *n (%)*	Double-rooted *n (%)*		Three-rooted *n (%)*			Total *n (%)*
		B	P	MB	DB	P	
Apical foramen location							
Centrally in the apex	104 (75.9%)	101 (91.0%)	99 (89.2%)	2 (100%)	2 (100%)	2 (100%)	310 (84.9%)
Laterally	17 (12.4%)	10 (9.0%)	12 (10.8%)	0 (0.0%)	0 (0.0%)	0 (0.0%)	39 (10.7%)
Both	16 (11.7%)	0 (0.0%)	0 (0.0%)	0 (0.0%)	0 (0.0%)	0 (0.0%)	16 (4.4%)
No. of apical foramina							
One	61 (44.5%)	106 (95.5%)	110 (99.1%)	2 (100%)	2 (100%)	2 (100%)	283 (77.5%)
Two	66 (48.2%)	5 (4.5%)	1 (0.9%)	0 (0.0%)	0 (0.0%)	0 (0.0%)	72 (19.7%)
Three	10 (7.3%)	0 (0.0%)	0 (0.0%)	0 (0.0%)	0 (0.0%)	0 (0.0%)	10 (2.8%)
Apical deltas							
Apical delta present	29 (21.2%)	3 (2.7%)	1 (0.9%)	0 (0.0%)	0 (0.0%)	0 (0.0%)	33 (13.2%)
Apical delta absent	108 (78.8%)	108 (97.3%)	110 (99.1%)	2 (100%)	2 (100%)	2 (100%)	332 (86.8%)

## Discussion

This is the first study in Yemen that evaluated root and canal morphology of permanent maxillary first premolar teeth. Several methods are used to investigate root canal morphology, including root sectioning, modeling, radiographic examination, tooth-clearing technique, cone-beam computed tomography (CBCT) and micro-computed tomography (micro-CT) imaging. Neelakantan et al. [22] compared the efficacy of four tomography methods with digital radiography and a tooth-clearing technique and concluded that only two tomography methods, CBCT and peripheral quantitative computed tomography, were as accurate as canal staining and tooth-clearing technique in identifying root canal systems. On the other hand, clearing technique was replaced with micro-CT technology which was proven to be the current reference method for the ex vivo study of the root canal anatomy. Micro-CT is preferred upon teeth clearing method due to the significant low detection of Vertucci type I canal in cleared teeth and fine anatomical details when compared to micro-CT method [23]. This limitation of teeth clearing method can be explained by incomplete diffusion of ink dye leading to distorted internal anatomy of cleared teeth and resulting in a different root canal type [23–25]. Although micro-CT has gained popularity because it provides accuracy, high resolution, and can be applied for detailed quantitative and qualitative measurements of the root canal anatomy, micro-CT is not available in all parts of the world, especially underdeveloped and developing countries. Moreover, the cost and radiation dose of micro-CT are other factors. In this study, canal staining and teeth clearing technique was used as suggested by Peiris [13], to determine the root canal morphology. Apart from being inexpensive and easy to conduct, other important advantages of clearing technique include retaining the original form of the canal, enabling the assessment of canal form and morphology with maintenance of the samples for long time [22].

Root canal morphology has been classified in different ways by several investigators in the literature [4, 26–30]. Weine et al. [26] classification includes four types depending on the pattern of division of the main root canal of a tooth along its course from the floor of the pulp chamber to the root apex. Meanwhile, Vertucci [4] categorized the root canal morphology in a more descriptive manner into eight types within three main groups. The first group includes three canal types (types I, II, and III), all with one apical foramen. The second one includes four canal types (types IV, V, VI, and VII), all exiting with two apical foramina. The third one includes the last canal type in this classification (type VIII) with three apical foramina. Gulabivala et al. [27, 28] developed two root canal classification systems that were based on observations of root canal configurations within mandibular molars in a sample of Burmese and Thai individuals, respectively. Additional types not present in Vertucci et al. classification were found. A different approach to root canal classification has been offered by Sert and Bayirli [29], who proposed a classification system differentiated by sex on the mandibular and maxillary permanent teeth among Turkish individuals. Fourteen new root canal configurations not included in other previous classification systems were described. Ordinola-Zapata et al. [31] used micro-CT imaging to evaluate of C-shaped mandibular first premolars in a Brazilian subpopulation. They reported several new anatomical variations and complexities of the root canal anatomy that were not included in previous classifications. Ahmed et al. [30, 32, 33] proposed new coding system for classifying root main and accessory canal morphology as well as teeth with anomalies to provide detailed information of the tooth and its root and canal anatomical features. In the present study, Vertucci classification [4] was used as reference because it is the most widely used classification in the literature and is still used in newly published papers [23, 34, 35]. Therefore, for the previous reasons, and also for easier results comparison, it was used in this study. However, additional root canal configurations [27–29] along with Vertucci classification were taken into consideration in this study.

Previous studies of the number of roots in maxillary first premolars showed various results. The prevalence of single-rooted maxillary first premolars (54.8%) in Yemeni population was in agreement with the findings of Pecora et al. [7], who reported that 55.8% of their specimens had one root. Walton & Torabinejad [36] referred to the existence of 50% of maxillary first premolars with two roots. In the present study, the prevalence of double-rooted premolars was in 44.4% of the specimens. The prevalence of three-rooted maxillary first premolars (0.8%) in this study was consistent with other studies performed in Turkish, Saudi, and Jordanian populations [8, 11, 12]. The number of roots of the maxillary first premolars as reported in previously mentioned studies and studies elsewhere [37–39] is summarized in Table 3 alongside the results of this study.

**Table 3:** In vitro studies on root morphology of the permanent first maxillary premolar

Author	Year	Population	Sample *(n)*	Single-rooted *(%)*	Double-rooted *(%)*	Three-rooted *(%)*
Vertucci & Gegauff	1979	North America	400	39.5	56.5	4
Walker	1987	China	100	60	40	0
Pecora et al.	1991	Brazil	240	55.8	41.7	2.5
Loh	1998	Singapore	957	49.4	50.6	0
Kartal et al.	1998	Turkey	300	37.3	61.3	1.3
Chaparro et al.	1999	Andalusia	150	40	56.7	3.3
Lipski et al.	2005	Poland	142	15.5	74	9
Atieh	2008	Saudia Arabia	246	17.9	80.9	1.2
Awawdeh et al.	2008	Jordan	600	30.8	68.4	0.8
Present study	–	Yemen	250	54.8	44.4	0.8

Vertucci & Gegauff [5] reported that maxillary first premolar was the only tooth which showed all eight types of Vertucci canal configurations. This was in accordance with this study in which all eight types of Vertucci canal configurations were found. In addition, in the current study, 2.8% of premolars showed types I, III, and IV of Gulabivala [27, 28] canal configurations, and 1.2% of Sert & Bayirli [29] additional canal types. More interesting findings of this study were the new root canal configurations found in eight premolars (3.2%). Although these canal types represent a low percentage but their treatment is challenging. Table 4 summarizes the percentage of root canal configurations in maxillary first premolars reported in previous studies [8, 10–13, 37–39] along with the results of the present study.

**Table 4:** In vitro studies on root canal configuration of the permanent first maxillary premolar

Author	Year	Population	Sample *(n)*	Vertucci’s root canal configuration *(%)*								Additional types *(%)*
				I	II	III	IV	V	VI	VII	VIII	
Vertucci & Gegauff	1979	North America	400	8	18	–	62	7	–	–	5	–
Caliskan et al.	1995	Turkey	100	3.9	5.9	–	78.4	5.9	5.9	–	–	–
Kartal et al.	1998	Turkey	300	8.7	1	–	71.3	14.7	2.3	0.3	1.3	–
Chaparro et al.	1999	Andalusia	150	1.3	37.3	–	58	–	–	–	3.3	–
Sert & Bayirli	2004	Turkey	200	10.5	12.5	5.5	61.5	3.5	1	–	3	–
Lipski et al.	2005	Poland	142	2.1	6.3	–	82.4	–	–	–	9.2	–
Peiris^a^	2008	Sri Lanka	153	1.3	16.3	2	64	5.9	5.9	0.7	–	3.9
	2008	Japan	81	4.9	29.6	2.5	45.7	2.5	8.6	–	–	6.2
Atieh	2008	Saudia Arabia	246	8.9	26.8	–	63	–	–	–	1.2	–
Awawdeh et al.	2008	Jordan	600	3.3	10.2	0.3	79.7	2	2.3	–	1.5	0.7
Weng et al.	2009	China	95	6.3	22.1	3.2	64.2	3.2	1	–	–	–
Present study	–	Yemen	250	13.2	4.4	8.0	55.6	5.6	1.6	3.6	0.8	7.2

^a^This study was performed in a Sri Lankan and Japanese populations

The occurrence of accessory canals in this study was 52.8% with maximum number noticed in the apical third (37.6%) of the roots. This was in accordance with text book of endodontics, where the highest incidence of accessory canals was found in the apical third of the root [40]. Different investigators [5, 8, 12, 29, 38, 39] have reported variations in the prevalence of accessory canals in maxillary first premolars (Table 5).

**Table 5:** In vitro studies on root canal morphology (accessory canals, inter-canal communications, apical foramina, deltas) of the permanent first maxillary premolar

Author	Year	Population	Sample ***(n)***	Accessory Canals ***(%)***	ICCs ***(%)***	Apical Foramina		Deltas ***(%)***
						Central ***(%)***	Lateral ***(%)***	
Vertucci & Gegauff	1979	North America	400	49.5^a^	34.2	12	88 ^a^	3.2
Caliskan et al.	1995	Turkey	100	33.3	17.7	33.3	66.7	21.6
Kartal et al.	1998	Turkey	300	26	7	15.3	84.7	7.7
Sert & Bayirli	2004	Turkey	200	33	12	34	76	30.7
Awawdeh et al.	2008	Jordan	600	19.3	7	60	40	4.3
Weng et al.	2009	China	95	51.7	–	–	–	–
Present study	–	Yemen	250	52.8	34.4	84.9	15.1	13.2

^a^Percentage is from canals number (788) not teeth number

Inter-canal communications (ICCs) or transverse anastomoses/isthmuses were present in 34.4% of the specimens with highest percentage being in the middle third of the root (19.2%). This was in agreement with textbook of endodontics, where highest incidence of ICCs was found in the middle third of the root [40]. An isthmus is a narrow, ribbon-shaped communication between two root canals that contains pulp or pulpally derived tissue. It functions as a bacterial reservoir. This communication is of clinical significance as it may be difficult to debride and fill adequately [41, 42]. The prevalence of ICCs in maxillary first premolars as reported in studies elsewhere [5, 8, 12, 29, 38] is summarized in Table 5 alongside the results of this study.

The location of apical foramen is of clinical significance during working length determination, which often depends on the average position of the apical constriction relative to the root apex [41, 42]. In the present study, the apical foramen was found to be central in 84.9% of the studied specimens. This is much higher than previous studies that showed that the apical foramen was centrally-located in 12 to 60% of their specimens (Table 5). The low percentage of laterally-located apical foramina in this study in comparison to other studies may be due to ethnicity. In addition, teeth used in the present study were collected from young patients attending orthodontic clinics. Obviously, age would mostly affect apical foramina location due to deposition of secondary dentine within the root canal that moves the site of the apical constriction away from the apex. These may explain why in this study the apical foramina were centrally-located in most studied specimens compared to previous studies [5, 8, 12, 29, 38] (Table 5). Apical deltas were observed in 13.2% of the collected Yemeni maxillary first premolars. The incidence of apical deltas in the maxillary first premolar as reported in previous studies [5, 8, 12, 29, 38] is summarized in Table 5 alongside the findings of this study.

## Conclusion

Yemeni permanent maxillary first premolars are mainly single-rooted and predominantly present type IV Vertucci canal morphology. The finding of additional canal configurations in this study is low but should be kept in mind when performing endodontic therapy for these teeth. The results of the present study further confirm the importance of a thorough knowledge of root canal morphology for each population and the need of a careful exploration and radiographic examination of these teeth prior to endodontic therapy.

## Abbreviations

CBCT: cone-beam computed tomography
ICCs: Inter-canal communications
MEC: Medical Ethics Committee
micro-CT: micro-computed tomography
